# Immunological Memory in Imiquimod-Induced Murine Model of Psoriasiform Dermatitis

**DOI:** 10.3390/ijms21197228

**Published:** 2020-09-30

**Authors:** Kevin Fenix, Danushka K. Wijesundara, Allison J. Cowin, Branka Grubor-Bauk, Zlatko Kopecki

**Affiliations:** 1 Basil Hetzel Institute for Translational Health Research, Discipline of Surgery, Adelaide Medical School, The University of Adelaide, Woodville South, Adelaide, SA 5011, Australia; kevin.fenix@adelaide.edu.au (K.F.); branka.grubor@adelaide.edu.au (B.G.-B.); 2 School of Chemistry and Molecular Biosciences, University of Queensland, Brisbane City, Brisbane, QLD 4072, Australia; d.wijesundara@uq.edu.au; 3 Future Industries Institute, University of South Australia, Mawson Lakes, Adelaide, SA 5095, Australia; allison.cowin@unisa.edu.au

**Keywords:** psoriasis, tissue-resident memory T cells, resolved lesions

## Abstract

Psoriasis is a common chronic inflammatory skin condition manifested by T cell responses and characterized by preferential recurrence at previously inflamed sites upon withdrawal of treatment. The site-specific disease memory in psoriasis has been linked to CD8^+^CD103^+^ tissue-resident memory T cells (Trm) in the epidermis which were previously thought to only provide “frontline” protection against pathogens and immunosurveillance during cancer development. In this study, we correlated the presence of a subset of the Trm cells which are also CD49a^+^ with disease severity in human psoriatic lesions with acute and chronic disease. Using an imiquimod (IMQ)-induced murine model of psoriasiform dermatitis, we also investigated the level of CD49a^+^ Trm cells in acute, chronic and resolved psoriatic lesions. Investigation of clinical human samples showed that patient disease severity highly correlated with the numbers of epidermal CD49a^+^ Trm cells. Additionally, this subset of Trm cells was shown to persist in resolved lesions of murine psoriasiform dermatitis once clinical disease features had subsided. Importantly, these CD49a^+^ Trm cells showed significantly higher levels of granzyme B (GzmB) production compared to acute disease, suggesting a potential role of CD49a^+^ Trm cells for psoriatic re-occurrence in resolved patients. Better understanding of epidermal CD49a^+^ Trm cell activity is necessary for development of advanced treatment strategies for psoriasis to permit long-term, continuous disease control.

## 1. Introduction

Psoriasis is a T lymphocyte-mediated systemic chronic skin disease affecting up to 3% of the population and more than 125 million people worldwide [1]. Although genetic and environmental factors greatly contribute to disease severity, disease pathogenesis is mainly driven by regulatory T cell imbalance leading to the activation of innate immune cells and pathogenic T cells, resulting in excessive skin inflammation and hyperproliferation of keratinocytes [2,3]. Interactive responses between infiltrating leucocytes, resident skin cells and an array of inflammatory cytokines and chemokines produced in the skin under the regulation of the cellular immune system governing disease progression [4]. Psoriatic lesions are highly vascular and densely infiltrated with T cells and dendritic cells, both of which are key regulators of disease pathogenesis. The hallmark of psoriasis is the relapsing nature of the disease and long-term persistence of lesions in the same anatomical regions (90% in the same location) and the appearance of new lesions in places where treated lesions have successfully resolved, often necessitating long-term therapies [5]. In the past decade, advancements in the development of psoriatic therapies and targeted biological drugs have significantly improved patient outcomes; however, disease relapse, alongside an understanding of the disease priming which results in lesion recurrence, is still lacking [6,7]. Site-specific immunological memory response in psoriasis has been linked to both CD8^+^ CD103^+^ tissue resident memory T cells (Trm) and dendritic cells in the epidermis [5]. Trm cells were previously thought to only provide “frontline” protection against infectious pathogens and immunosurveillance during cancer development; however, recent studies have shown clear involvement of epidermal Trm cells in site-specific T-cell-driven disease memory involved in psoriasis pathogenesis [8]. Epidermal Trm cells have been shown to be retained in resolved psoriatic lesions of patients and mediate detrimental pro-inflammatory responses by producing interleukin (IL)-17a and IL-22 cytokines upon stimulation, both of which are critical in psoriasis pathogenesis [8,9]. A number of studies have suggested that Trm cell biology is critical in disease recurrence as their resistance to elimination by current conventional therapies provides a potential mechanism to resume the psoriatic inflammatory feedback loop at the site of residence [7].

Trm cells are a critical and unique component of the adaptive immune system, with specific roles in orchestrating downstream innate and adaptive responses upon reactivation [10]. They are most known for their roles in barrier function in mucosal tissue and the skin; however, they possess phenotypic variation between tissue types. Therefore, a better understanding of Trm biology is required to optimize their generation, maintenance and function during disease pathogenesis or clinical relapse. Trm cells are characterized by surface proteins that regulate their localization and contribute to their development and/or survival, namely E-cadherin binding CD103 (integrin αE paired with β7) and collagen binding CD49a (integrin α1 paired with β1) with a preference for type IV collagen in the lamina densa underlying the epithelium [11]. Both integrins have been implicated in the formation of Trm cells, and while CD49a has been implicated in regulating T cell migration and survival via its interaction with collagen IV, our understanding of these receptors in regulating interactions with the tissue and T cell motility is incomplete and underappreciated [10]. Collagen binding integrins have previously been shown to regulate tissue inflammation in various disease states [12,13] and studies using integrin α1 knockout mice have shown a key role for integrin α1 in the development of psoriasis [14] and contact hypersensitivity [15] in different dermatitis models. Blocking of CD49a has been shown to augment T cell migration into the epidermis and subsequent immune responses in a xenotransplant model of psoriasis, thus highlighting the role of CD49a in local surveillance mechanism of the Trm population and, more specifically, the immunopathology of psoriasis [10,14]. Additionally, CD103 function in vivo has been shown to support the initial accumulation of epidermal T cells while CD49a has been shown to be important in the long-term persistence of epidermal Trm cells [10].

To date, many studies have shown that CD49a-negative Trm cells in psoriatic skin contribute to IL-17 production and disease severity. However, it is now clear that CD49a expression defines distinct functional subsets of Trm cells that produce interferon (IFN)-γ and cytolytic granules, such as perforin and GzmB, suggesting a mechanism for priming of psoriatic skin and a role for Trm cells in disease relapse as effectors in secondary lesion development [9]. Indeed, freshly isolated CD49a^+^ Trm have been shown to display a transcriptional profile indicative of cytotoxic function [16]. Recent studies have shown that epidermal Trm cells capable of triggering psoriasis from tissue responses accumulate from non-lesional skin of psoriatic patients [17]. Additionally, these cells are retained in the skin of patients with resolved psoriasis and can produce cytokines critical for psoriasis pathogenesis, hence providing a site-specific T-cell-driven disease memory in psoriasis [8]. Despite advances in the understanding of Trm cell function in psoriatic disease, limited responses have been observed in mouse models of disease due to the methods employed to quantify cellular responses. In this study, we set out to investigate the correlation between CD49a^+^ Trm cells with disease severity in human psoriatic skin and investigate if the imiquimod-induced mouse model of psoriasiform dermatitis could be useful for analysis of Trm levels in acute, chronic and resolved psoriatic lesions. A further understanding of Trm cell biology and pathogenic activity, as well as the mechanism of activity in psoriasis, is required in order to drive the safe design of therapeutic approaches that manipulate or eliminate pathogenic Trm cells without losing local immunity [18].

## 2. Results

### 2.1. Levels of Epidermal Trm Cells Closely Correlate with Disease Severity in Human Psoriatic Patients

**Figure 1:**
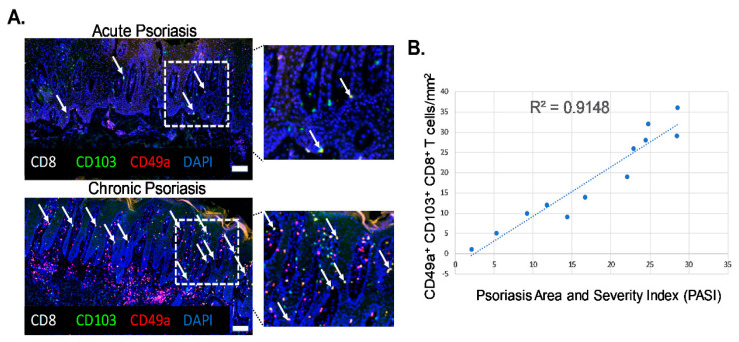
Levels of epidermal CD49a^+^ Trm cells closely correlate with disease severity in human psoriatic patients. (**A**) Representative immunohistochemistry images of acute and chronic inflamed psoriatic lesions stained with markers of CD49a^+^ Trm cells (white arrows). *n* = 6/group. Magnification ×20, insert white boxed region ×40. Scale bar = 50 µm. (**B**) Graphical analysis of correlation between Trm cell number and Psoriasis Area and Severity Index (PASI) score. *n* = 6/group.

Biopsies of actively inflamed psoriatic lesions were collected from twelve patients of both sexes with an average age of 58.4 years and from various body locations including the scalp, abdomen and extremities. Histological analysis of samples revealed characteristics associated with plaque psoriasis including acanthosis, elongated rete pegs, hyperkeratosis and perivascular lymphocytic infiltration. Patients were grouped into acute or chronic groups based on clinical presentation and history of disease, degree of histological inflammation and Psoriasis Area and Severity Index (PASI) score. Patients with a PASI score of below 18 were considered as acute and those with a PASI score of above 18 were considered chronic. Psoriatic lesions from human patients were analyzed for numbers of CD49a^+^ Trm cells (Figure 1A, white arrows, CD8^+^ CD103^+^ CD49a^+^, yellow cells). CD49a^+^ Trm cells mainly localized at the basement membrane, allowing interaction with E-cadherin and type IV collagen. The numbers of CD8^+^ CD103^+^CD49a^+^ Trm cells correlated with patient PASI scores, revealing a high degree of correlation between the number of CD49a^+^ Trm cells and patient severity, with a greater number of CD49a^+^ Trm cells present in more chronic severe disease (R^2^ = 0.9148; *p* = 0.0002) (Figure 1B).

### 2.2. Imiquimod (IMQ)-Induced Murine Model of Psoriasiform Dermatitis Shows Clinical Features of Acute, Chronic and Resolved Inflammation

**Figure 2:**
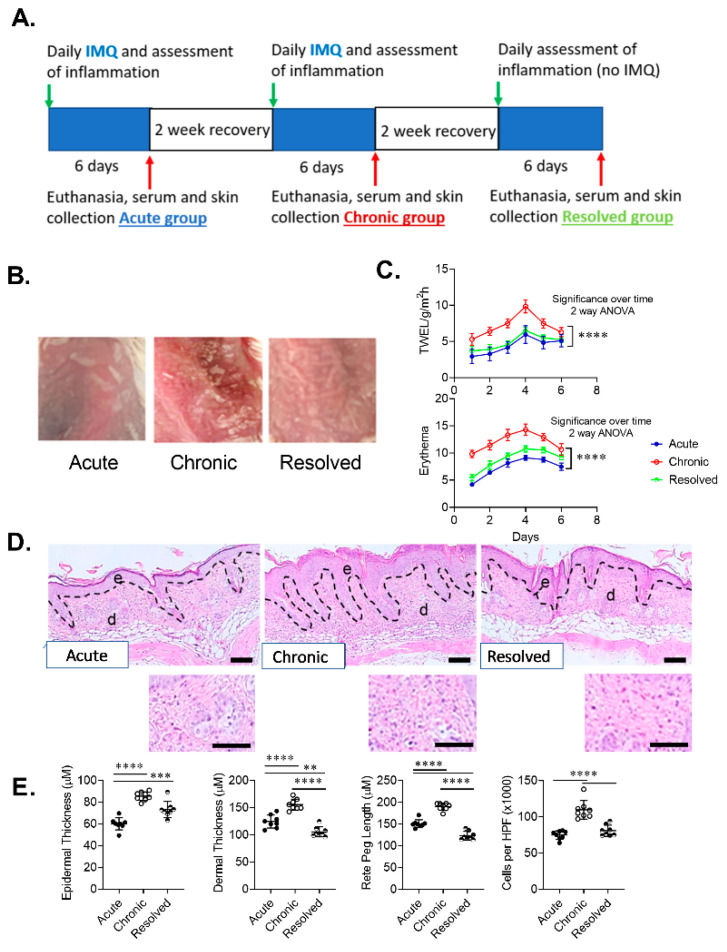
Development of imiquimod-induced acute, chronic and resolved psoriasis-like dermatitis in mice skin. (**A**) Model timeline. (**B**) Representative macroscopic images of developed imiquimod-induced psoriasis-like dermatitis. (**C**) Graphical analysis of transepidermal water loss (TEWL) and erythema measurements in mice with acute, chronic and resolved psoriasis-like dermatitis. (**D**) Representative sections of psoriasis-induced mice skin stained with hematoxylin and eosin (scale bar = 100 µm, inset scale bar = 25 µm). (**E**) Graphical analysis of psoriasis-like mouse skin in acute, chronic and resolved groups comparing histological changes including epidermal thickness, rete peg length, dermal thickness and a total number of inflammatory cells per high-power field (HPF) (original magnification ×1000). Data are presented as mean ± SD. *n* = 8/group. ** *p* < 0.01, *** *p* < 0.001, ****p < 0.0001.

The IMQ mouse model of psoriasiform dermatitis is one of the most utilized animal models in psoriasis research and has phenotypical features of human psoriasis [19]. Daily application of IMQ cream over a period of 6 days results in increased immune response resulting in erythema, swelling and scales [20]. While this model is widely used, to the best of our knowledge, this model has only been used to study acute and chronic forms of psoriasis and no previous studies have utilized this model to examine Trm cell levels in acute, chronic and resolved psoriasis-like dermatitis. To achieve acute, chronic and resolved psoriasis, we used 10-week-old BALB/c female mice, eight mice in each group. The acute group received one 6-day cycle of IMQ application. The chronic group received two 6-day cycles of IMQ application with a two-week recovery period between IMQ cycles. The resolved group followed the chronic group protocol with an additional 2-week and 6-day recovery period (Figure 2A). All groups showed a clear development of inflammation during each cycle of IMQ application. Representative images of developed psoriasis-like dermatitis and skin scaling for each treatment group show clear evidence of skin inflammation, which was most pronounced in mice with chronic psoriasis-like disease (Figure 2B). The severity of induced psoriasis-like dermatitis was determined in each group by analysis of skin barrier function and erythema measurements. Both transepidermal water loss (TEWL) measurements and spectrophotometric measurements of skin redness showed increasing impairment in skin barrier function and erythema during each assessment period. Using a two-way ANOVA with multiple comparisons post-test, the chronic group showed significantly increased levels of TEWL and skin erythema when compared to acute and resolved groups, while no significant difference in TEWL and a small but significant difference in erythema was observed between acute and resolved groups over a six-day period (Figure 2C).

A hallmark of psoriasiform dermatitis is epidermal hyperplasia, which was evident in all treatment groups and was most pronounced in the chronic group. Histological analysis of psoriasis-like dermatitis showed thickened epidermis including increased perivascular lymphocytic infiltrate in the upper epidermis in all groups (Figure 2D) compared to non-lesional skin (data not shown). Examination of the epidermal thickness of the lesional skin showed that mice in the acute group had significantly thinner epidermis compared to chronic and resolved groups. Dermal thickness and length of rete pegs were slightly higher in acute than resolved skin while the amount of cellular inflammatory infiltrate in psoriasis-like skin showed no significant difference. In contrast, histological analysis of the chronic group showed significantly thicker dermis, length of rete pegs and inflammatory cell infiltrate, thus demonstrating higher disease severity (Figure 2E). Thus, each treatment group in our developed model had a unique pathology that mimicked those observed in the clinic.

### 2.3. Epidermal Trm Cells Remain in Murine Skin of Resolved Psoriasiform Dermatitis and Demonstrate Cytotoxic Activity

**Figure 3:**
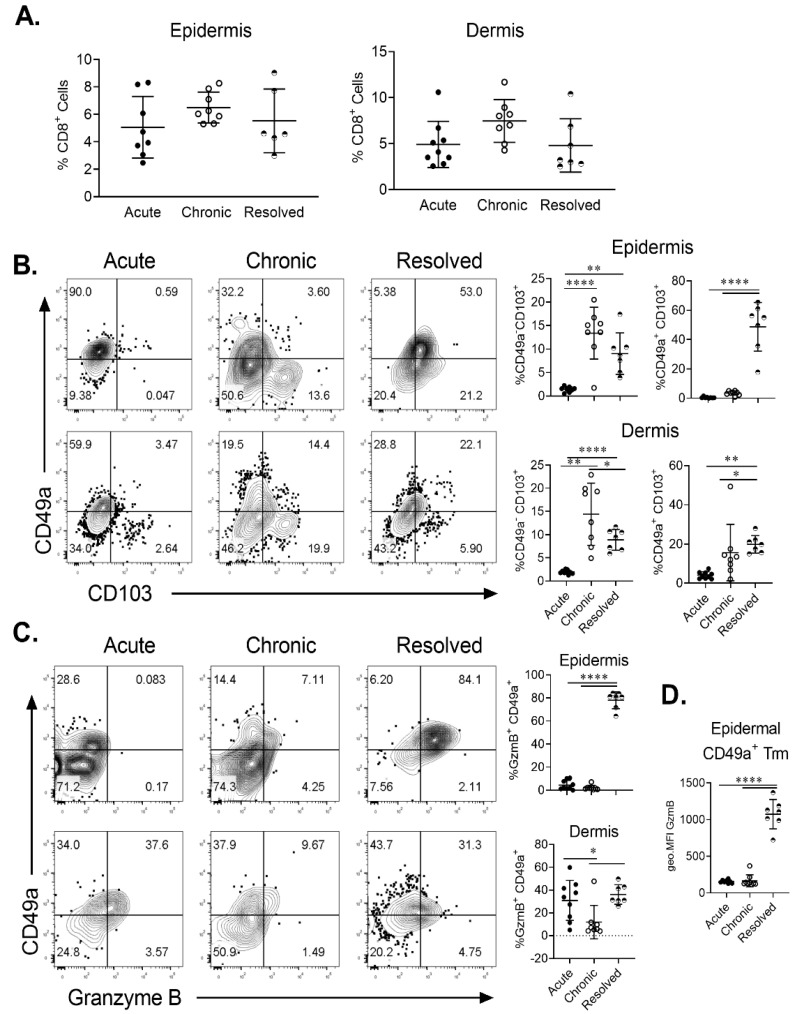
Fluorescence-activated cell sorting (FACS) analysis of Trm cell levels in skin of imiquimod-induced acute, chronic and resolved psoriasiform dermatitis. (**A**) Levels of CD8^+^ T cells in the dermis and epidermis of psoriasis-like murine skin expressed as percentage of live cell. (**B**) Representative FACS plots of CD49a vs. CD103 staining on CD8^+^ T cells found in the epidermis (top) or dermis (bottom). Proportion of CD49a^+^ CD103^+^ and CD49a^−^ CD103^+^ Trm cells was quantitated for each condition. (**C**) Representative FACS plots of CD49a vs. GzmB staining on CD103^+^ CD8^+^ T cells found in the epidermis (top) or dermis (bottom). The proportion of GzmB expressing CD49a^+^ Trm cells was quantitated. (**D**) Quantitation of GzmB geometric mean expression by epidermal CD49a^+^ Trm cells. Each dot represents one mouse skin sample (*n* = 8/group). Data are presented as mean ± SD. * *p* < 0.05, ** *p* < 0.01, **** *p* < 0.0001.

**Figure 4:**
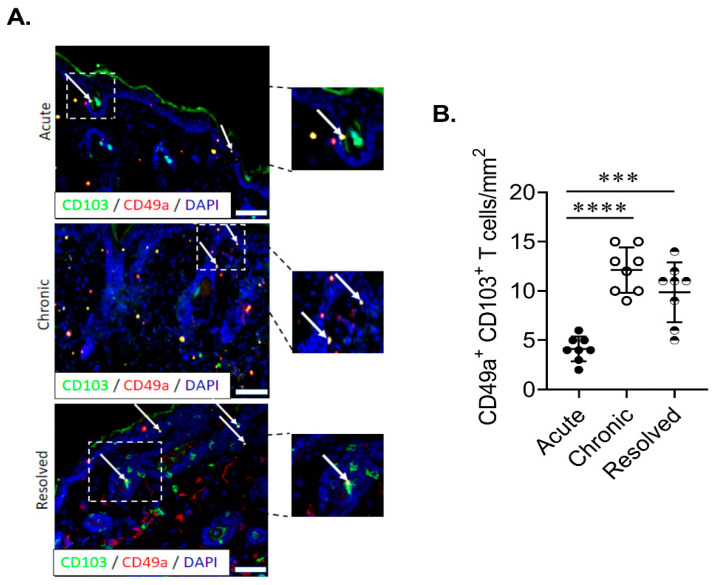
Epidermal Trm cells remain elevated in the murine skin of resolved psoriasiform dermatitis. (**A**,**B**) Representative images and quantitation of CD103^+^CD49a^+^ Trm cells in the epidermis of mice with acute, chronic and resolved psoriasiform dermatitis. Each dot represents one mouse skin sample (*n* = 8/group). Data are presented as mean ± SD. *** *p* < 0.001, **** *p* < 0.0001.

**Figure 5:**
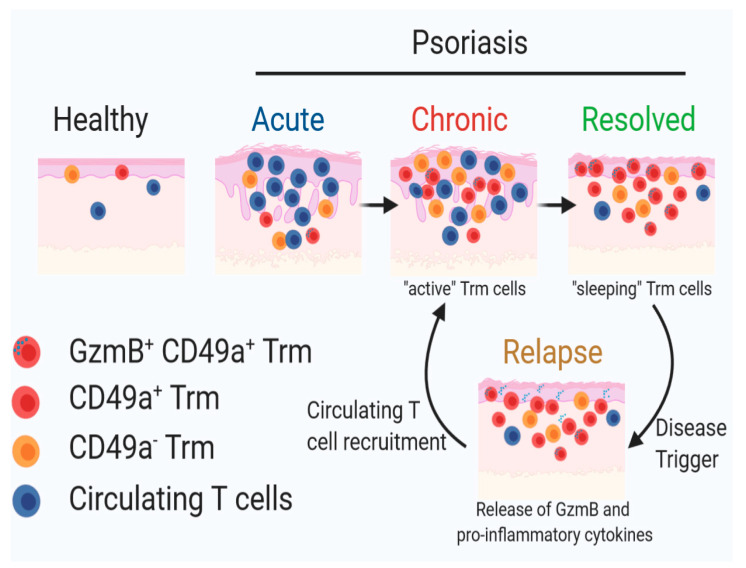
A schematic diagram of our proposed mechanism illustrating the role of CD49a^+^ Trm cells in clinical relapse underpinning psoriasis pathogenesis. Image was generated using BioRender.

In order to ascertain if site-specific Trm cells are present in IMQ-induced acute, chronic and resolved psoriasiform dermatitis in murine skin, we examined the population of total CD8^+^ and CD8^+^ CD103^+^ T cells in the dermis and epidermis. Flow cytometric analysis showed that CD8^+^ T cells were found in both the epidermis and dermis in all treatment groups (Figure 3A). Using CD49a/CD103 co-staining, we identified the two previously described Trm populations based on CD49a expression. Trm cells were significantly enriched in the chronic and resolved groups (Figure 3B). Interestingly, in the epidermis, the proportion of CD49a^-^ Trm cells was highest for the chronic psoriasiform dermatitis group, while CD49a^+^ Trm cells were enriched in the resolved group (Figure 3B). Examining CD49a/granzyme B (GzmB) co-expression on CD103^+^ Trm cells confirmed that cytotoxic activity was specific for the CD49a^+^ population. Of note, GzmB expression was highest within the epidermal CD49a^+^ Trm cells in the resolved group (Figure 3C). Measurement of relative GzmB expression using geometric mean fluorescence intensity on epidermal CD49a^+^ Trm cells showed that CD49a^+^ Trm cells in the resolved group produced significantly higher levels (Figure 3D) compared to acute and chronic groups. Significantly increased numbers of CD49a^+^ Trm cells in the epidermis of resolved groups vs. acute disease group were further confirmed using immunohistochemistry (Figure 4A,B). Taken together, the results show that CD49a^+^ Trm cells accumulate in psoriatic skin regardless of the current disease state (chronic vs. resolved); however, there is a clear increased accumulation of cytotoxic CD49a^+^ Trm cells in the resolved state, and we hypothesize that this accumulation of GzmB in CD49a^+^ Trm cells is critical in disease relapse (Figure 5).

## 3. Discussion

A major therapeutic challenge in treating patients with psoriasis is the high degree of disease relapse that occurs at previously affected sites once the treatment subsides owing to site-specific memory T cells. Studies in patients with clinically healed psoriasis have shown that resolved psoriatic lesions have a molecular fingerprint composed of over 250 gene products that are not fully normalized post-treatment, including both inflammation-associated and skin-structure related genes [21]. Clinical remission is therefore often short, with active inflammation detected 12–15 weeks post-treatment withdrawal. Highly activated epidermal T cells that survive the treatment have been implicated as driving this clinical relapse [8]. Dissecting different T cell populations present within the different anatomical compartments of the skin may lead to better understanding of disease pathogenesis and lead to more targeted therapies aimed at long-term disease management. T cell receptor sequencing has shown that in resolved psoriasis, pathogenic T cell clones are maintained at similar levels to those found in acute lesions [22]. Moreover, pathogenic T cell clones that remain in resolved, clinically healed lesions that are actively producing proinflammatory cytokines have been suggested to be Trm cells and their renewed activation results in keratinocyte activation, chemokine release and recruitment of circulating leukocytes, closely correlating with clinical relapse after therapy [7,23]. Interestingly, pathogenic Trm cells have also been shown to be present in the non-lesional skin of psoriatic patients [17]. Epidermal tissue resident memory T cells (Trm) offer a site-specific memory in psoriasis, while the dermal T cells have been shown to circulate between the resolved skin and the blood [8]. In this study, we focused on the subset of epidermal Trm cells that are CD49a^+^ as they have been recently shown to produce proinflammatory IFN-γ and play a role in cytotoxic activity underpinning disease relapse [9]. To the best of our knowledge, this is the first study to provide a correlation between Trm levels and patient disease severity and quantify Trm cell levels and cytotoxic function in acute, chronic and resolved lesions of murine skin using an imiquimod-induced model of psoriasiform dermatitis.

Research to date has indicated that CD49a and CD103 are not merely markers of Trm cells but also confer substrate specificities in their binding to collagen and E-cadherin as well as functional roles in site-specific disease memory [10,11]. In agreement with previous reports, we observe that CD49a^+^ Trm cells are present in both the epidermis and dermis in human psoriasis in both acute and chronic disease. It is well established that the development of the stable and resting epidermal T cell population occurs over time once the inflammation is largely resolved; however, we now demonstrate a high degree of correlation between disease severity and numbers of CD49a^+^ Trm cells in human acute and chronic psoriasis. While our study is limited by the ethical constraints in the collection of biopsy samples from patients with resolved psoriasis, our findings suggest that epidermal CD49a^+^ Trm cells may be useful as prognostic markers of disease severity or treatment efficacy. This could be particularly useful for several systemic biologic therapies under development. As it is currently unclear if administered antibodies penetrate the epidermis, screening for epidermal CD49a^+^ Trm cells in biopsies of psoriatic patients as prognostic markers of disease severity could inform disease response to treatment or potential for disease relapse in chronic difficult-to-treat patients which show limited success with treatment strategies or have higher incidence of disease recurrence. Additionally, this highlights a future area of research interest in developing multimodal targeted therapeutic approaches using both systemic therapies that target signalling pathways, as well as topical treatments that target Trm cells as a strategy to prevent clinical disease relapse in previously affected areas of the skin.

While the mechanisms surrounding the site-specificity of recurrent inflammation in human psoriasis are still being understood, animal models of disease that accurately represent clinical features of psoriasiform dermatitis offer opportunities for a better understanding of T cell biology in disease pathogenesis. We now show that the IMQ-induced model of acute, chronic and resolved psoriasiform dermatitis in mice skin offers a unique opportunity to study different phases of the inflammatory process associated with psoriasis and specifically immune responses that lead to disease relapse associated with enriched epidermal Trm cell subsets in areas of resolved disease. In agreement with previous studies, we show that a repeated daily administration of IMQ over a six-day period in mice leads to acute development of the disease [20] and that 2-week recovery periods between each IMQ administration cycle allow for the development of chronic and resolved disease states in respect to T cell levels and Trm populations observed in human studies [8]. Hence, we have developed a model where the contribution of CD49a^+^ Trm cells could be dissected in disease relapse.

Previous studies have characterized the phenotypical features of Trm cells in non-lesional, lesional and healthy psoriasis [24] and demonstrated the cytotoxic function of CD49a^+^ Trm in human skin [9]. Studies have also shown that Trm cells in psoriatic patients had specific properties not found in Trm cells of healthy skin, suggesting that lasting control of psoriasis disease may require suppression of Trm cells [22]. Additionally, human studies comparing the effects of different treatments on Trm levels in psoriasis have indicated that the overall epidermal T cell population is decreased in areas of resolved psoriasis; however, CD49a^+^ Trm cells remain enriched compared to healthy and non-lesional skin [8]. In agreement with these studies, using flow cytometry and immunohistochemistry, we demonstrate that CD49a^+^ Trm cells remain elevated in murine skin of resolved psoriasis-like disease, suggesting the formation of a stable and resting population of epidermal Trm cells in resolved psoriasis. Differences observed in epidermal CD49a+ Trm cell numbers in chronic disease between flow cytometry and immunohistochemistry are reflective of differences in analysis of whole back skin vs. individual biopsy/mouse. Additionally, in agreement with human studies which demonstrated a cytotoxic function of Trm in healed psoriasis [9], we now demonstrate a significantly elevated number of pathogenic epidermal Trm cells that secrete GzmB, suggesting that their cytotoxic activity has direct clinical relevance and should lead to future investigations of their function in disease relapse. Previous studies using human lesional and non-lesional psoriatic skin have demonstrated the contribution of GzmB^−^ cells to skin inflammation and disease pathogenesis [25]. Our observation of elevated GzmB^+^ CD49a^+^ Trm in lesions of resolved psoriasis suggests that these cells may play an important function in the in situ activation of epidermal T cells. Thus, this leads to tissue-specific re-occurrence of flare-ups and subsequent, more severe lesions in areas of skin previously affected by psoriatic lesions where there is a high probability of disease relapse, a common phenomenon observed in relapsing clinically resolved psoriatic patients. Additionally, previous human studies have shown that resolved psoriasis lesions retain expression of a subset of disease-related genes [21] and our study further supports the idea of Trm cell function in the activation of epidermal T cells and disease relapse.

Future studies in our group will evaluate the true pathogenic potential of Trm subsets using the IMQ-induced acute, chronic and resolved psoriasiform dermatitis model described in this study. Additionally, it would be interesting to further dissect the interplay between CD49a^+^ Trm cells and epidermal Langerhans cells in disease relapse as dysfunction of these cells has been attributed to the rapid initiation of inflammation in the same location, hence contributing to the memory of psoriatic eruptions [26]. Additionally, Langerhans cells are found in close proximity to T cells and have been shown to cross-talk with both T cells and keratinocytes, suggesting their potential role in renewed activation of T cells and disease recurrence [7]. A recent study has shown that Langerhans cells isolated from resolved psoriatic lesions have the ability to produce proinflammatory IL-23 post stimulation, further implying the potential role of these cells in restarting the activation of “sleeping” Trm cells, potentially the GzmB^+^ CD49a^+^ Trm cells described, leading to re-occurrence of psoriatic lesions (Figure 5) [26]. Previous studies have rightfully investigated the role of IL-17 in the pathobiology of this disease. While CD103^+^ Trm cells have the capacity to express IL-17, a large proportion of epidermal CD103^+^ T cells in human psoriatic patients are known to express CD49a that have potentially a more cytotoxic function rather than cytokine release, including in psoriatic “molecular fingerprints” that remain after disease treatment [7]. Due to the lack of a good animal model for this chronic inflammatory skin disease, the importance of this subset has not been proven. Here, we describe an imiquimod-induced psoriasiform dermatitis regimen that mimics human psoriatic resolved lesions, by showing increased epidermal CD49a^+^ Trm cells in clinically resolved murine skin.

## 4. Materials and Methods

### 4.1. Human Tissue Samples

Human psoriatic skin biopsies (*n* = 12) were obtained from the SA Pathology archive following approval from the Royal Adelaide Hospital Research Ethics Committee (HREC/12/RAH/190, 29/01/2016). The work described has been carried out in accordance with the code of ethics of the World Medical Association (Deceleration of Helsinki). Informed consent was obtained for experimentation with human samples and the tissue samples and clinical history and PASI score were deidentified for experiments and analysis. Patient confidentiality was strictly maintained, and the identities of the tissue samples and clinical data were only known to the principal investigator and the pathologist. Patient diagnoses were based on clinical features and histological characteristics of plaque psoriasis as confirmed by the examining pathologists. Patients with mild–moderate inflammation, PASI score of less than 18 and clinical disease of less than 5 years were grouped into the acute psoriasis group while patients with a high degree of inflammation, PASI score of more than 18 and clinical disease of more than 5 years were considered to have chronic psoriasis (*n* = 6/group).

### 4.2. Animal Studies

Mice were maintained according to the Australian code for the care and use of animals for scientific purposes under the protocols approved by the Child Youth and Women’s Health Service Animal Ethics Committee (AEC1094/07/2018, 20/07/2018). Ten-week-old female wild-type mice with the BALB/c background were used in this study. Psoriasiform dermatitis was induced in mice following a widely used protocol [19]. Dosing regimen of IMQ administration, recovery time between different groups (acute, chronic and resolved) and assessment of skin inflammation is illustrated in Figure 2A. During each cycle of imiquimod (IMQ) administration, mice received a daily topical application of a dose of 62.5 mg IMQ cream (5%) (Aldara, 3M Pharmaceuticals, St Paul, MN, USA) on the shaved dorsal skin for six consecutive days (daily dose of 3.125 mg of active compound). Mice were examined daily for cutaneous inflammation and the dorsa were photographed. The level of skin barrier impairment was assessed daily during each assessment cycle by transepidermal water loss measurements using a handheld vapometer (Delfin Technologies, Kuopio, Finland), following the manufacturer’s instructions. The level of skin erythema was measured daily during each assessment cycle using the DermaLab Combo (Cortex Technology ApS, Hadsundm, Denmark). The mice were killed after each IMQ cycle/assessment cycle (acute group day 6, chronic group day 26, resolved group day 46) (*n* = 8/group) and serum and skin collected for assessment of inflammation using histology and immunohistochemistry and for isolation of T cells for flow cytometry using established protocols [27].

### 4.3. Histology, Immunohistochemistry and Confocal Microscopy

Cryopreserved and paraffin-embedded fixed human and animal tissue samples were stained with hematoxylin and eosin or subjected to antigen retrieval and immunohistochemistry following routine protocols. Immunohistochemistry on paraffin-embedded fixed patient psoriatic lesions was performed by heat-induced antigen retrieval using 10 mM sodium citrate buffer, followed by blocking with Protein Block Serum-Free (Dako, Australia) for 1 h at RT in a humidified chamber. Primary antibodies against anti-human CD8 (1:50, CD/144B, Abcam), CD103 (1:250, EPR4166(2), Abcam) and CD49a (1:200, PA5-95563, ThermoFisher Scientific, Waltham, MA, USA) were applied and slides were incubated at 4 °C overnight before application of species-specific secondary antibodies anti-mouse IgG Alexa Fluor 647 (1:200, Jackson ImmunoResearch, West Grove, PA, USA), anti-rabbit IgG CY3 (1:200, Jackson ImmunoResearch, West Grove, PA, USA) and goat anti-rat Alexa Flour 488 (1:200, Invitrogen, Carlsbad, CA, USA) for 1 h at room temperature. DAPI was then added to the slide and they were incubated in the dark for 15 min. Finally, slides were mounted in Fluorescence Mounting Medium (Dako, Sydney, Australia). For cyrosectioned murine skin psoriatic lesions, slides were first fixed using ice-cold acetone for 10 min at −20 °C, followed by a similar staining process to that described above using anti-mouse CD8b.2 (1 in 100, 53-5.8, Biolegend, San Diego, CA, USA), CD103 (1:100, 2E7, Biolegend, San Diego, CA, USA) and CD49a (1:100, REA493, Miltenyi Biotec, Bergisch Gladbach, Germany) with anti-Armenian hamster IgG Alexa Fluor 488, anti-rat IgG CY3 and anti-human IgG Alexa Fluor 647 (all 1:200, Jackson ImmunoResearch, West Grove, PA, USA) used as secondaries. For verification of staining, non-specific binding was determined by omitting primary or secondary antibodies. All control sections had negligible immunofluorescence. Images were acquired using a LSM700 laser scanning confocal microscope or an Axio Scan Z.1 immunofluorescence slide scanner with Zen software (Zeiss, Adelaide, Australia). Integrated fluorescence intensity and cell counting was determined using AnalySIS software package (Soft-Imaging System GmbH, Munster, Germany). Negative controls and isotype control antibody were included to demonstrate antibody staining specificity. Control samples underwent the exact same staining procedure outlined except that the primary or secondary antibody was omitted. All control sections had negligible immunofluorescence.

### 4.4. Preparations of Skin Cell Suspensions and Flow Cytometry

Whole-skin biopsies were incubated in 5 U dispase (Life Technologies, Melbourne, Australia) overnight at 4 °C and epidermis was separated from dermis following established protocols. Epidermis was cut and incubated in trypsin (0.025%)/EDTA (0.01%) (Life Technologies) for 15 min and single-cell suspensions were prepared by pipetting. Dermis was incubated in collagenase III (3 mg/mL; Worthington, Pan Biotech, Aidenbach, Germany) for 90 min with DNAse (5 mg/mL) in 10% FCS RPMI 1640 media and further processed using a cell strainer. Single-cell suspensions were then stained in a 96-well round bottom plate at 2 × 10^6^ lymphocytes per well. Cells were first resuspended with Fixable Viability Stain 620 (1:500, BD) for 15 min at RT, followed by washing with FACS Buffer (1% FCS/0.05% Sodium Azide/PBS). Cells were resuspended in 50 uL of FACS Buffer and blocked with FC Block (BD) for 10 min at RT. Antibody cocktail containing anti-mouse CD8a APC-Cy7 (1:100, 53-6.7, BD), CD103 BV510(1:50, M290, BD) and CD49a PE(1:100, HA31/8) was then added directly on top and incubated for 30 min at 4 °C. Cells were then permeabilized using Cytofix/Cytoperm^TM^ kit (BD Biosciences, North Ryde, Australia) for 30 min at 4 °C, then washed twice in BD Perm/Wash buffer before staining with granzyme B Alexa Fluor 647 (1:100, GB11, BD Biosciences, North Ryde, Australia) for 30 min at 4 °C. Samples were washed with BD Perm/Wash buffer before finally being resuspended in FACS Buffer and acquired using a BD FACSCANTO II^T^ (BD Biosciences, North Ryde, Australia) Data were analyzed using FlowJo^TM^ V10 (BD Biosciences, North Ryde, Australia).

### 4.5. Statistics

The results are presented as mean ± SD. Data analysis was performed using GraphPad Prism 8 (Graphpad, CA, USA) with statistical significance between different groups assessed by one-way or two-way analysis of variance (ANOVA) plus a Tukey’s multiple comparisons post-test when comparison between more than 2 groups was required. A value of *p* < 0.05 was set for the significance value. Pearson’s correlation coefficient was used for analysis of PASI correlation with Trm cell levels and calculation of coefficient of determination.

## 5. Conclusions

The imiquimod-induced murine model of acute, chronic and resolved psoriasiform dermatitis offers a robust opportunity to study Trm cell function in dermal and epidermal skin of psoriasis-like disease. Data presented here confirm earlier studies of epidermal Trm cells remaining elevated in psoriasis post resolution of clinical features. This study suggests that the cytotoxic activity of a subset of CD49a^+^ epidermal Trm cells may contribute to recurrent episodes of inflammation leading to recurrent disease development at sites of previously inflamed skin. Future research aimed at better understanding epidermal Trm cell cytotoxic activity will lead to the development of advanced treatment strategies for psoriasis which may allow long-term, continuous disease control.

## Abbreviations

GzmB	granzyme B
IMQ	imiquimod
PASI	Psoriasis Area Severity Index
Trm	tissue-resident memory T cells
